# Endocrine Side Effects in Patients Treated with Immune Checkpoint Inhibitors: A Narrative Review

**DOI:** 10.3390/jcm12155161

**Published:** 2023-08-07

**Authors:** Nicia I. Profili, Roberto Castelli, Antonio Gidaro, Alessandro Merella, Roberto Manetti, Giuseppe Palmieri, Margherita Maioli, Alessandro P. Delitala

**Affiliations:** 1 Department of Medicine, Surgery and Pharmacy, University of Sassari, 07100 Sassari, Italy; 2 Department of Biomedical and Clinical Sciences Luigi Sacco, University of Milan, Luigi Sacco Hospital, 20157 Milan, Italy; 3 Department of Biochemical Science, University of Sassari, 07100 Sassari, Italy

**Keywords:** immune checkpoint inhibitors, endocrine side effects, PD-1, PD-L1, CTLA-4, thyroid, adrenal, pituitary

## Abstract

Checkpoint inhibitors are monoclonal antibodies that elicit an anti-tumor response by stimulating immune system. Their use has improved the treatment of different types of cancer such as melanoma, breast carcinoma, lung, stomach, colon, liver, renal cell carcinoma, and Hodgkin’s lymphoma, but several adverse events have been reported. Although the etiology of these effects is not completely understood, an uncontrolled activation of the immune system has been postulated. Indeed, some studies showed a cross reactivity of T cells, which acted against tumor antigens as well as antigens in the tissues of patients who developed immune-related adverse events. Despite the known possibility of developing immune-related adverse events, early diagnosis, monitoring during therapy, and treatment are fundamental for the best supportive care and administration of immune checkpoint inhibitors. The aim of this review is to guide the clinician in early diagnosis, management, and treatment of the endocrinological adverse effects in the major endocrine glands (thyroid, pituitary, adrenal, endocrine pancreas, and parathyroid).

## 1. Introduction

Cancer cells can create a perfect tumor microenvironment by changing their surface antigens, thus evading immunosurveillance. In addition, tumor cells express ligands that bind inhibitory T-cell receptors, known as immune checkpoints, that deactivate T cells [1]. Immune checkpoints are specific proteins that physiologically balance the immune destruction of foreign antigens and the development of autoimmune host organ injury [2]. Peripheral tolerance is a physiological process aimed at suppressing potentially autoreactive naïve T cells in lymph nodes or in peripheral tissues and is regulated by two different pathways: the immune checkpoints cytotoxic T-lymphocyte-associated antigen 4 (CTLA-4) and programmed death-1 (PD-1) [3].

CTLA-4 is a protein receptor expressed by activated T cells and regulatory T cells. Activation of T cells requires the binding of major histocompatibility complex (MHC) I and II receptors to tumor-associated antigens presented by antigen-presenting cells (APCs) and the binding of the CD28 receptor to CD80 and CD86 (B7 ligand subtypes) on the APCs [4]. These signals result in amplification of the immune response. T-cell activation causes an upregulation of CLTA-4, which competes with CD28 for CD80 and CD86 binding on APCs. This, in turn, can downregulate the T-cell activation, with a decreased immune response to tumor-associated antigens [5].

PD-1 is a protein expressed on the surfaces of activated T cells, B and NK cells, macrophages, dendritic cells, and monocytes [6] and has a pivotal role in the immune response. Indeed, PD-1 promotes the apoptosis of specific T cells in lymph nodes and reduces apoptosis in regulatory T cells. However, it also acts as a pro-tumorigenic factor in cancer cells. Indeed, programmed death ligand 1 (PD-L1) signaling is inappropriately activated in many cancers by binding to its receptor and promoting the activation and the survival signaling pathway in tumor cells. PD-1 has two binding ligands: PD-L2 and PD-L1; the latter is usually expressed by the same cells (except NK cells and monocytes) in inflammatory conditions [7]. In addition, PD-L1 is expressed by cancer cells, and some clinical studies have reported a direct association between its expression on cancer cells with tumor size, lymph node involvement, and overall survival [8]. 

Immune checkpoint inhibitors (ICI) are monoclonal antibodies that elicit an anti-tumor response by stimulating immune system. They act by targeting inhibitory receptors and ligands expressed on T lymphocytes and tumor cells. These drugs can effectively overwhelm the cancer cell’s resistance by allowing immune cells to target and remove them. Mechanisms of action change along with the type of drug. The anti-CLTA-4 antibody prevents CD80 and CD86 on an APC from binding to CTLA-4 on T cells. Blocking CLTA-4 signaling, this drug enhances T-cell activation and the proliferation of the immune response. On the other hand, anti-PD-1 and anti-PD-L1 antibodies block PD-L1, inhibiting the interaction between PD and PD-L1.

The use of ICIs, in both monotherapeutic and combined modes [9], has improved the treatment of different types of cancer, including melanoma, breast carcinoma, lung, stomach, colon, liver, renal cell carcinoma, and Hodgkin’s lymphoma (Table 1).

However, several adverse events have been reported that are caused by the uncontrolled activation of the immune system and are known as immune-related adverse events (irAEs) [10,11]. The pathogenesis of endocrine irAEs was not clear [12]; however, the most prevalent hypothesis accepted an interplay between genetics and cellular and humoral autoimmunity [13]. Indeed, studies showed a cross reactivity for T cells, which acted against tumor antigens as well antigens in tissue where the irAEs developed. In addition, T cells are further stimulated by specific chemokines that are found to be increased in patients with irAEs, who also have circulating autoantibodies against organ-specific targets. Finally, an association with the HLA-DR allele has been also documented for some irAEs. 

In this review, we describe the epidemiology, pathophysiology, diagnosis, and treatment of the irAEs developed in the thyroid, gland pituitary, adrenal gland, pancreatic βcells, and parathyroid gland. Due to the lack of appropriate reports, our narrative review did not include the pineal gland or gonads (ovary and testis).

## 2. Thyroid

Thyroid disorders are the most common endocrine irAE after ICI treatment (Table 2).

Hyperthyroidism is defined as increased thyroid hormone synthesis and secretion, whereas thyrotoxicosis refers to the clinical syndrome of excess circulating thyroid hormones, irrespective of the source [58]. Regardless of the presence of dysthyroidism or its clinical manifestation, they share the same biochemical diagnosis, which is characterized by reduced or suppressed thyrotropin (TSH). The further evaluation of free thyroxine (FT4) and free triiodothyronine (FT3) allows us to distinguish subclinical disease (FT4 and FT3 within the respective reference range) and overt disease, which is characterized by increased FT4 and FT3. Patients treated with ICIs may develop thyrotoxicosis, which two possible causes recognized: destructive thyroiditis and autoimmune hyperthyroidism (e.g., Graves’ disease). On the other hand, overt hypothyroidism is diagnosed in the presence of increased TSH and reduced FT4 and FT3, which are within the reference range in the subclinical disease. The development of hypothyroidism after ICI treatment can be secondary to a destructive thyroiditis or due to an autoimmune origin. Overall, hypothyroidism seems to be more frequent than thyrotoxicosis (6.6% vs. 2.9%) [59]; however, previous studies have reported variable frequencies for irAEs, which are mostly dependent on the different timings of the assessment of thyroid hormones, the definition of the diseases, and the class ICI prescribed.

The exact pathogenetic mechanism is not completely understood. It is not clear whether PD-L1 molecules are expressed in healthy thyroid glands, but previous reports showed that these ligands are expressed during inflammation [60]. In addition, whereas recent studies suggested a critical role of cytotoxic memory T cells in the pathogenesis of destructive thyroiditis induced by anti-PD-1 antibodies, no studies have analyzed the role of anti-CTLA-4 antibodies.

The pathologic features of the thyroid specimens of patients treated with ICIs did not clarify the exact pathogenetic mechanism of thyroid disorders related to ICI treatment. Indeed, some studies reported typical findings of autoimmune thyroiditis, such as lymphohistiocytic aggregates, lymphocytes and histiocytes positive for CD163, and a predominance of CD8+ T cells [61,62]. However, the same studies described other features that are not typical of Hashimoto’s thyroiditis: abundant clusters of necrotic cells, rare/absent thyrocytes, and granulomas and destruction of follicles.

Mouse models have allowed the possibility of understanding the possible mechanisms of thyroid disorders after ICI treatment. One study reported that anti-PD-1 administration caused destructive thyroiditis that could be prevented by preceding depletion of CD4+ cells. Flow cytometric analyses showed that central and effector CD4+ cell levels were higher in the peripheral blood of patients who developed destructive thyroiditis, as compared with those without destructive thyroiditis [63].

Finally, an association with HLA-DPA1x01:03 and HLA-DPB1x02:01 alleles has been documented [64].

### 2.1. Hyperthyroidism

The frequency of hyperthyroidism changes along with the type of treatment: patients treated with anti-PD-1 have an increased frequency of hyperthyroidism compared with those treated with anti-CLTL-4 and anti-PD-L1, 3.2% vs. 1.7% and 0.6%, respectively [65]. Combination therapy with anti-PD-1 and anti-CLTL-4 antibodies increases the frequency of its onset to 8% [65]. The median time of onset for hyperthyroidism was 7 weeks for Graves’ disease and earlier for destructive thyroiditis (6 weeks) [66]. Early- and late-onset cases have been also described [67]. The type of drug (anti-CTLA-4 or anti-PD-1) does not affect the time of onset of hyperthyroidism [68], which has a median duration of 36 days. The onset of hyperthyroidism is earlier in patients on combination therapy. Indeed, 83% of hyperthyroidism cases were diagnosed within 21 days from the beginning of the treatment, whereas 100% of cases were diagnosed within 84 days. Patients treated with a monotherapy developed hyperthyroidism later. Indeed, 36% and 90% of cases were diagnosed within 21 and 84 days, respectively, from the beginning of the ICI [69]. Although thyrotoxicosis can be divided into destructive thyroiditis and Graves’ disease, the latter is rare and only three reports have been clearly documented so far [70,71,72]. The symptoms of thyrotoxicosis are usually mild and not specific, which is consistent with the low grade of biochemical severity. However, cases of symptomatic thyroid storm have been also reported [73].

Recovery after hyperthyroidism was reported in up to 57% of cases.

However, it should be noted that the terms thyrotoxicosis and hyperthyroidism are often used interchangeably, thus producing a possible altered estimate of their frequencies. In addition, the thyrotoxic phase is often self-limited and asymptomatic, thus leading a possible underestimation of the phenomenon.

### 2.2. Hypothyroidism

The frequency of hypothyroidism is higher in patients treated with anti-PD-1 (7%), but cases have also been reported during anti-CTLA-4 (3.8%) and anti-PD-L1 (3.9%) treatments. A higher frequency (13.2%) was reported after combination therapy with anti-PD/anti-CLTL-4 [65].

Hypothyroidism usually develops 6–12 weeks after the beginning of ICI therapy [67,74], although hypothyroidism with a precedent thyrotoxic phase seems to have an earlier presentation [67,75]. In addition, 60% of the patients with thyrotoxicosis subsequently developed hypothyroidism after a median time of 42 days, whereas none of the patients with hypothyroidism developed hyperthyroidism. Cases of hypothyroidism without prior thyrotoxicosis regimen therapy (mono or combination therapy) did not affect the time of onset in hypothyroid patients [75]. The predictors of the development of autoimmune thyroid disorders have not been completely elucidated and the role of thyroid autoantibodies in the context of irAEs have not been completely understood. Indeed, it is not clear whether these antibodies are the causative agent of the dysfunction or whether they are an epiphenomenon secondary to the released thyroid antigens during a destructive thyroiditis [76].

The symptoms of hypothyroidism are not specific, but cases of myxedema crisis have been also reported [77].

### 2.3. Predictors of irAEs

Some studies have tried to identify predictive biomarkers for the development of irAEs in the thyroid gland. Among these, TSH is the biomarker that has been most extensively studied. Indeed, an increased baseline TSH level in patients showed a positive association with the development of thyroid dysfunction when the TSH level was above 5 uIU/mL [78]. Other studies have also reported that a high-normal level of TSH increased the risk for developing hypothyroidism [79]. The same authors suggested a cut-off value of 1.67, and other studies reported similar findings [80,81]. It should be noted that thyroid autoimmunity, a major cause of both hyperthyroidism and hypothyroidism, is commonly found in the general population and its presence at baseline represents a risk factor for the development of irAEs [70]. Previous studies have reported that patients with a high titer of antibodies against thyroglobulin (TGAb) and antibodies against thyroperoxidase (TPOAb) before the beginning of checkpoint inhibitor treatment had an increased risk of developing thyroid dysfunction. However, at the same time, patients negative for TGAb and/or TPOAb at baseline may develop a disorder of the thyroid gland. Indeed, some studies have reported that IR thyroid disorders are more frequent in patients with preexisting autoantibodies against thyroid antigens (TGAb and/or TPOAb) compared with those who were negative at baseline [82], whereas other studies failed to report this association [83]. Another study reported the trend of peripheral blood lymphocytes in a subject who developed hypothyroidism after nivolumab treatment. The patient, who had positive TPOAb and TGAb, developed hypothyroidism thereafter and displayed an increase in thyroid autoantibodies. Follicular helper T cells increased from baseline after 2 weeks (from 0.9% to 3.1%) and decreased to 1.2% after 4 months from the beginning of nivolumab treatment. Thus, the increased proliferation of follicular helper T cells may promote the development of thyroid autoantibodies [84]. The interpretation of the levels of these biomarkers is somewhat tricky. Indeed, a slightly increased level of TSH at baseline has been reported to be a risk factor for the development of an irAE. However, it should be noted that patients with such TSH values are also more prone to develop overt hypothyroidism, regardless of the use of an ICI. In addition, most of the studies reported a single TSH quantification, thus potentially including individuals with nonthyroidal illness or a transient abnormality. Similar limitations can be noted for thyroid autoantibodies. Indeed, their fluctuating levels, which can become also negative, do not allow discrimination of their contribution to the onset of irAEs. In addition, the different results reported by the studies do not allow us to discern whether they are linked to irAEs or an innocent epiphenomenon.

Some studies have also reported that subjects who develop endocrine irAEs have a longer progression-free survival [85,86]; however, this effect is not reported by other studies [87]. Although the causes of this association are still not clear, the type of cancer as well as the presence of preexisting thyroid diseases did not affect the relationship. However, it should be noted that the study by Chmielewska et al. also reported that the median progression-free survival in patients without endocrine adverse events was shorter than patients with endocrine events (2 months vs. 9 months), thus suggesting that occurrence of endocrine complications significantly reduced the risk of disease progression (HR = 0.415, *p* < 0.05) [87]. Similar results were reported by other groups [88].

The treatment of thyroid side effects allows patients to continue being treated with ICI drugs, which would need to be withdrawn in the presence of other irAEs (for example acute hepatitis). Studies have reported that therapy resumption in patients who experienced moderate to severe irAEs increased the risk of developing new irAEs [89]. One study reported that ICI rechallenge caused the onset of hyperthyroidism in 1.3% of patients, which was more prevalent than the initial irAEs before the discontinuation of the drug [90]. 

### 2.4. Management

The guidelines suggest checking TSH and FT4 levels at baseline, prior to any ICI treatment. Thereafter, TSH and FT4 should be assessed closely at each course of treatment for 6 months, every 2–3 courses for the following 6 months, and then monitored every 6 months [91]. Additional tests can be evaluated clinically upon their results. Indeed, in cases of hypothyroidism, TPOAb can be tested, whereas in cases of hyperthyroidism it is suggested to check the TSH receptor antibody. In cases of hyperthyroidism with normal TSH receptor antibody, FT3 and imaging exams (ultrasonography and scintigraphy) may help for the differential diagnosis [92].

Treatment is based on clinical and biochemical findings and in cases of inconsistency between them, drug interferences should be suspected [93]. The guidelines identify a grading system for hyperthyroidism, based mainly on symptoms such as weight loss, rapid heartbeat, increased sweating, and irritability [94]. Grade 1 includes asymptomatic patients or those with mild symptoms. Grade 2 identifies patients with moderate symptoms that are able to perform the activities of daily living. Grade 3 includes patients with severe symptoms that are unable to perform the activities of daily living. The treatment of Graves’ disease is based on antithyroid drugs, in particular in cases of persistent hyperthyroidism (>6 months), whereas most cases of mild thyrotoxicosis can be managed with a short-term treatment with beta blockers (e.g., propranol 20 mg bis in die) to relieve the symptoms of thyrotoxicosis [91]. A role for high doses of glucocorticoids has been suggested [95], but there is no clear evidence for its preventive use [96]. The use of high doses of glucocorticoid therapies is recommended only in cases of severe thyrotoxicosis and severe ophthalmopathy [91]. TSH levels and presence of symptoms (constipation, weight gain, hair loss, extreme tiredness, or weakness) allows identification of three levels of hypothyroidism grading: grade 1 occurs when TSH < 10 mUI/mL and there is an absence of symptoms, grade 2 is represented by the presence of moderate symptoms and TSH persistently > 10 mUI/mL, and the patients have grade 3–4 if symptoms are severe and there is an inability to perform the activities of daily living [94]. This grading system allows identification of which patients need specific therapies. Patients with grade 1 do not need treatment with levotyroxine, which is mandatory for grade 2 and grade 3–4 [91]. A recent study showed that patients with hypothyroidism secondary to ICI treatment required higher doses of levothyroxine [97].

## 3. Pituitary

There are two main types of irAEs at the pituitary level: isolated adrenocorticotropic hormone deficiency and hypophysitis, which is characterized by pituitary enlargement and a deficiency in multiple anterior pituitary hormones This classification, proposed by some authors, could be misleading because isolated corticotropic deficiency could be simply an unrecognized hypophysitis. Indeed, it is common to find isolated pituitary hormone deficiency secondary to different causes. For example, rare cases of isolated post-hypophysitis caused by the COVID-19 vaccine [97] have been reported as well as cases of hypophysitis [98]. The exact pathophysiology of these associations, as well their cause–effect relationship still needs to be elucidated. 

After thyroid disorders, hypophysitis is the second most common immune-mediated side effect. Although diagnosis of thyroid dysfunction is easy to perform, the detection of hypophysitis is challenging because it is based upon a combination of clinical symptoms, hormone secretion abnormalities, and radiological findings. The frequency of hypophysitis is higher in patients treated with monoclonal antibodies against CTLA-4 (1–18%) [99,100,101,102,103] than those treated with PD-1 inhibitors (0.5–1.5%) [103,104,105]. An older age and the male gender have been described as risk factors in one small series [106].

The onset of hypophysitis induced by immune checkpoint inhibitors seems to be related to the type of drug used. Indeed, the median onset time is shorter in patients treated with ipilimumab or combination therapy compared with those receiving anti-PD1 antibodies (9.3 weeks and 12.5 weeks vs. 25.8 weeks, respectively,) [107]. The recovery of the pituitary–adrenal axis after ICI withdrawal is extremely rare [108].

The pathophysiology of hypophysitis related to irAEs is not completely understood. A direct toxicity mechanism has been proposed, but a type II hypersensitivity reaction seems the most probable mechanism [109]. Pituitary antibodies have been documented in a recent study, which suggested that their positivity at baseline and after treatment could become predictive biomarkers for pituitary dysfunction. Another interesting hypothesis was proposed by Caturegli et al., who analyzed at autopsy the pituitary glands of cancer patients treated anti-CTLA-4 therapy, one of which had clinical and pathologic evidence of hypophysitis [110]. The authors demonstrated that the CTLA-4 antigen was expressed in all pituitary endocrine cells but that the highest level was found in the patient with severe hypophysitis. This finding suggested that high levels of CTLA-4 antigen in the pituitary can cause a necrotizing form of hypophysitis through type IV and type II immune mechanisms [111].

The induction of arginine vasopressin deficiency (AVP-D) by immune checkpoint inhibitors is very rare. A recent study by Bai et al. reported the onset of AVP-D in 16 out of 6089 patients (0.003%); the condition developed as an endocrine side effect secondary to immune checkpoint inhibitor treatment [112]. Another recent review reported 11 cases of AVP-D [113]. Among these, in 5 out 11 patients the side effect was diagnosed in the context of panhypophysitis induced by ipilimumab, both as single agent and in combination with an anti-PD-1. Four patients developed isolated AVP-D. Another case was secondary to hypothalamitis induced by anti-PD-L1, and the last case was diagnosed in a patient who developed concomitant metastasis in the anterior pituitary. Although typical symptoms were present in all patients (polyuria, polydipsia), it should be noted that the diagnosis of AVP-D was not convincing in some cases. Indeed, the water deprivation test was performed only in two patients and magnetic resonance scans were available in 10/11 patients. In addition, specific treatment with desmopressin was performed in 5/11 patients. Interestingly, the immune checkpoint inhibitor was discontinued in 7/11 patients, two patients had a delayed administration, and two patients continued specific treatment. The pathogenesis of AVP-D related to immune checkpoint inhibitors is unknown and should be evaluated in the context of its onset. Indeed, the presence of AVP-D secondary to the presence of hypophysitis may be explained by the inflammation and edema in the pituitary stalk. On the other hand, isolated AVP-D should have a different etiology, probably due to an autoimmune origin that causes selective damage to the posterior pituitary. This hypothesis is strengthened by the well-known autoimmune pathogenesis in idiopathic AVP-D. However, even in this latter disease the exact pathophysiology is still not clear. A pathogenetic role for autoantibodies against rabphilin-3A has been hypothesized [114]. Another pathogenetic hypothesis is the presence of necrotizing small-vessel vasculitis, which is often associated with the positivity of antineutrophil cytoplasmic antibodies (ANCA) [115]. Thus, the use of immune checkpoint inhibitors might cause isolated AVP-D by increasing the risk of developing an autoimmune disorder, similarly to what happens in cases of idiopathic AVP-D.

### Management

A diagnosis of hypophysitis should be suspected on clinical evaluation (headache, polyuria, or fatigue) and based on pituitary magnetic resonance. Hormonal tests are mandatory to evaluate any deficiency.

Treatment of diagnosed or suspected adrenal crisis is mandatory with 100 mg hydrocortisone followed by 50 mg quater in die, whereas standard doses of glucocorticoid replacement therapy are suggested after the acute event [91]. Gonadal hormone replacement therapy is suggested in specific cases and is important for wellbeing and to prevent the complications of hypogonadism. Testosterone replacement treatment is appropriate in men with persistent and confirmed hypogonadotropic hypogonadism, whereas estroprogestinic therapy is suggested in women of reproductive age. In both sexes, the absence of contraindications is mandatory before starting any hormonal replacement therapy. AVP-D treatment is based on desmopressin, which controls diuresis and avoids an imbalance of osmolality and natremia, similar to the management of other etiologies [115]. The guidelines suggest against treating growth hormone deficiency in the presence of an active malignancy [91].

## 4. Adrenal

Primary adrenal insufficiency related to ICI therapy is a very rare complication and is characterized by deficiencies of glucocorticoids and mineralocorticoids. Only a few cases have been reported so far [66,116]. Tan et al. reported six cases of primary adrenal insufficiency [66]. Males were mostly affected (mean age of 52 years). Anti-PD-1 was used in 4/6 patients, whereas 2/6 patients were treated with ipilimumab. Three out six patients had a melanoma. The authors reported that the onset of symptoms started 10 weeks after the initiation of checkpoint inhibitor therapy. All patients were benefited by specific treatment with steroid supplementation therapy. No previous studies documented a spontaneous recovery after the cessation of ICIs.

The exact pathophysiological mechanism is largely unknown, but the available data seem to suggest two different mechanisms: inflammatory adrenalitis and an autoimmune origin. The latter seems somewhat more likely and is supported by findings reported in the studies. Indeed, two studies reported anti-adrenal autoantibodies [117,118] and one reported adrenal atrophy, similar to the findings reported in patients with Addison’s disease [117]. However, other cases had normal adrenal imaging. Interestingly, 50% of patients with primary adrenal insufficiency had an additional endocrinopathy. Patients usually complained of symptoms of adrenal insufficiency from 2 weeks up to 8 months after ICI treatment.

### Management

The diagnosis of primary adrenal insufficiency is based on the guidelines [119] and requires basal adrenocorticotropic hormone (ACTH) and cortisol. In cases of equivocal results, the low dose synacthen test can be used to demonstrate the adrenal insufficiency, which requires a cortisol level below 485 nmol/L after 60 min. Physicians should be aware of the concomitant use of corticosteroids. Screening for autoantibodies against 21-hydroxylase is also advisable [91]. Patients affected by primary adrenal insufficiency related to ICI therapy need the same treatment as adrenal insufficiencies of other origin [118]: cortisone acetate 20–30 mg daily or hydrocortisone 15–25 mg daily associated with fludrocortisone 0.05–0.15 mg.

## 5. Type 1 Diabetes Mellitus

Type 1 diabetes mellitus is an uncommon irAE that was reported in less that 1% of patients in a 6-year period [119]. The clinical onset may have a wide range of days from the initial administration of the ICI (from 13 to 504 days) but is most frequently within 90 days [120]. Symptoms of hyperglycemia are common and 85% of patients have ketosis, whereas ketoacidosis is less common and reported in up to 70% of patients.

Anti-PD-1 and anti-PD-L1 antibodies cause new onset type 1 diabetes mellitus more than anti-CTLA-4. Melanoma was the most frequent form of cancer associated with the onset of diabetes mellitus. Incidence rates of the disease vary among studies and the type of drug used and have been reported to be in the range 0.3–3.5% [121].

Similar to other irAEs, the exact underlying mechanism which leads to type 1 diabetes is not clear. One study suggested that low expression of PD-1 may increase T-cell proliferation and activation in patients with acute onset type 1 diabetes, thus causing beta-cell destruction [122]. This hypothesis is strengthened by the findings of islet infiltration with T lymphocytes after ICI administration [123].

Islet-related autoantibodies have been found in type 1 diabetes related to anti-PD1 antibodies, but frequency is variable and ranges from 5% to 50% [120,124]. However, the exact relationship between their presence and the pathophysiology of the disease remains unclear. Nevertheless, autoantibodies seem to a have a predictive role for the development of type 1 diabetes. Indeed, 40% of patients who tested positive for islet-related autoantibodies developed diabetes after ICI treatment [125].

### Management

The diagnosis of type 1 diabetes related to ICI therapy is based on international guideline. In cases of the confirmation of hyperglycemia, patients should be tested for urine ketone, blood pH, and specific autoantibodies (against glutamic acid decarboxylase, islet antigen-2, insulin, and the zinc transporter ZnT8). The standard treatment required insulin injection.

## 6. Parathyroid

Primary hypoparathyroidism is the rarest endocrine effect among the irAEs; it has been documented in only six cases in the scientific literature [120]. On the contrary, a finding of hypocalcemia is more frequent but could be mostly related to the reduction of albumin, which is often found in cancer patients. The cases of hypocalcemia related to primary hypoparathyroidism were symptomatic (paresthesia, weakness, fatigue) and the laboratory findings were consistent with the clinical suspicions (low calcium and low/undetectable parathormone). Due to the anecdotical reports of this association, it is not possible to have an estimate of frequency for the drugs related to its onset. Nivolumab, ipilimumab, and pembrolizumab, as monotherapies or in combination therapies, have been associated with the onset of primary hypoparathyroidism.

Normal parathyroid tissue does not normally express PD-1/PD-L1 or CLTA-4, but a possible autoimmune nature for the disease has been suggested by the finding of autoantibodies activating the calcium-sensing receptor [126].

### Management

The diagnosis of primary hypoparathyroidism is supported by the findings of hypocalcemia, which must be corrected for albumin values, and low or undetectable parathormone. After diagnosis, patients should be treated with calcium and calcitriol, as used in hypoparathyroidism due to other causes.

## 7. Conclusions

ICI treatment has dramatically increased the life expectancy of cancer patients. However, their use has been associated with several irAEs, most of which affected endocrine glands. Hypothyroidism is, overall, the most prevalent disease reported in patients treated with anti-PD1 antibodies who developed a prior destructive thyroiditis, which could be asymptomatic and unknown. The periodical follow-up of cancer patients is mandatory to avoid the onset of symptomatic clinical disease, which can also be a sudden onset, such as an acute primary adrenal insufficiency.

## Figures and Tables

**Table 1:** Monoclonal antibodies against CLTA-4, PD-1, and PD-L1.

Drug Name	Ig Isotype and Physiological Function
Ipilimumab	Target: CTLA-4
	Ig isotype: IgG1
	Physiological function: Depleting Treg cells
Tremelimumab	Target: CTLA-4
	Ig isotype: IgG2
	Physiological function: Neutralizing inhibitory signal in T cells
Pembrolizumab	Target: PD-1
	Ig isotype: IgG4
	Physiological function: Neutralizing inhibitory signal in T cells
Nivolumab	Target: PD-1
	Ig isotype: IgG4
	Physiological function: Neutralizing inhibitory signal in T cells
Cemiplimab	Target: PD-1
	Ig isotype: IgG4
	Physiological function: Neutralizing inhibitory signal in T cells
Dostarlimab	Target: PD-1
	Ig isotype: IgG4
	Physiological function: Neutralizing inhibitory signal in T cells
Atenolizumab	Target: PD-L1
	Ig isotype: IgG1
	Physiological function: Neutralizing inhibitory signal in T cells
Durvalumab	Target: PD-L1
	Ig isotype: IgG1
	Physiological function: Neutralizing inhibitory signal in T cells
Avelumab	Target: PD-L1
	Ig isotype: IgG1
	Physiological function: Neutralizing inhibitory signal in T cells; Antibody-dependent cellular cytotoxicity

Abbreviations: CTLA-4, T-lymphocyte-associated antigen 4; PD-1, Programmed death-1 (PD-1); PD-L1, Programmed death-1 ligand; Ig, Immunoglobulin; IgG, Immunoglobulin type G.

**Table 2:** Frequency of thyroid disorders according to the immune checkpoint inhibition: recent studies.

Author	Anti-PD-1		Anti-PD-L1		Anti-CTLA-4	
	Hyperth	HypoT	HyperT	HypoT	HyperT	HypoT
Lu et al. [14]	20.2%	66.5%	28.8%	62.0%	27.9%	55.3%
Hu et al. [15]	10.3%	47.7%				
Xu et al. [16]	14.3%	7.1%				
Akturk et al. [17]		3.8% *			0.0%	0.0%
Ueba et al. [18]	4.8%	6.3%	9.5%	3.6%		
Huang et al. [19]		4.3%				
Schulz et al. [20]		18% **				
van Laar et al. [21]	13.7%	19.6%				
Qu et al. [22]			0.9%	12.8%		
Zhao et al. [23]		8.3%				
Liu et al. [24]		76.7%				
Hiraoka et al. [25]				6.7%		
Lu et al. [14]	22.7% **	62.0% **				
Uhara et al. [26]		14%				
Wu et al. [27]		27.5%				
Baek et al. [28]	8.2%	20.6%	9.5%	14.3%		
Schonfeld et al. [29]	17.5% **	0.3%				
Labadzhyan et al. [30]		23.4%				
Kim et al. [31]		14.8%				
Phillips et al. [32]		19% **				
Sonehara et al. [33]		12.4% **				
Marabelle et al. [34]		11.6				
Duan et al. [35]		8.7%				
O’Malley et al. [36]	7.1% ***	14.2% ***				
Fuereder et al. [37]		40.9%				
Ngamphaiboon et al. [38]	2.7% **	7.5% **				
Fidilio et al. [39]	46.2% **	44.5% **				
Zhang et al. [40]		14.8%				
Yu et al. [41]		24.2%				
Mayer et al. [42]		7% ***				
Yamamoto et al. [43]		5.7%				
Chan et al. [44]	6.2% **	17.9% **				
Makker et al. [45]		54.3%				
Trullas et al. [46]		12.4%				
Muir et al. [47]	31.0% ***	8.0% ***				
Yoon et al. [48]		16.7%		9.7%		
Griewing et al. [49]		28.0% *				
Alkrekshi et al. [50]		2.5% **				
Leddon et al. [51]		19.1% *				
McDermott et al. [52]		14.5%				
de Azevedo et al. [53]				17.3%		
Almutairi et al. [54]		56.2%				17.8%
Zayas-Soriano et al. [55]		25.7% *				
Robert et al. [56]	3.0%	9.1%				
Zhou et al. [57]	7.9%	13.1%				

Abbreviations: PD-1, Programmed death-1; PD-L1, Programmed death-1 ligand CTLA-4, T-lymphocyte-associated antigen 4; HyperT, Hyperthyroidism; HypoT, Hypothyroidism. * Anti-PD-1 or anti-PD-L1. ** Anti-PD-1 and/or anti-PD-L1 and/or anti-CTLA-4. *** Anti-PD-1 or anti-CTLA-4.

## Data Availability

Not applicable.
